# Effect of fibrin-rich plasma and collagen sponge on healing of the palatal mucosa

**DOI:** 10.4317/jced.60549

**Published:** 2023-07-01

**Authors:** Rocío Alvarez-Medina, María E. Guerrero, Nancy E. Córdova-Limaylla, Luis E. López-Llamosas, José L. Huamaní-Echaccaya, Jerson J. Palomino-Zorrilla, José C Rosas-Díaz

**Affiliations:** 1 School of Stomatology, Universidad Privada San Juan Bautista, Lima, Perú; 2 Department of Medico Surgical Stomatology, Faculty of Dentistry, Universidad Nacional Mayor de San Marcos, Lima, Perú; 3 School of Stomatology, Universidad Privada San Juan Bautista, Ica, Perú

## Abstract

**Background:**

The purpose was to evaluate the variation in thickness and early healing of the donor area of the palate with the placement of a collagen sponge and the use of fibrin-rich plasma (L-PRF).

**Material and Methods:**

Thirty patients who required mucogingival surgery treatment were selected and distributed into 2 groups. After obtaining the free palate graft, L-PRF was placed in Group A, and a collagen sponge was placed in Group B. The healing process of the palate was evaluated at 24 hours and 7, 14, 21 and 28 days postsurgery. The thickness of the donor area (palate) was evaluated using an acrylic splint. These measurements were made before and 4 months after surgery.

**Results:**

In the collagen sponge group, less gain of the palatal mucosa was observed, with a mean difference of 0.1 ± 0.8 mm (CI: −0.341–0.518) (*p*=0.691), whereas in the fibrin-rich plasma group, a mean difference of 0.0 ± 0.5 mm (CI: −0.229–0.229) (*p*=0.934) was found; however, when comparing the gain of the palatal mucosa in both groups, no significant difference was observed (*p*=0.932). The healing index at 24 hours indicated the presence of clots, on Day 28 vascularisation and total epithelialisation (100.0%), and finally, the collagen sponge group on Day 14 presented 93.3% partial vascularisation of connective tissue and 33.3% L-PRF (*p*=0.001).

**Conclusions:**

There was no statistically significant difference in the thickness of the palatal mucosa after the use of L-PRF and the collagen sponge.

** Key words:**Palate thickness, connective tissue graft, fibrin-rich plasma, collagen sponge, palate healing.

## Introduction

Currently, an increasing number of patients present with loss of gingival tissue around teeth that affects both aesthetics and function. Among mucogingival deformities and conditions, the most common are a lack of keratinized gingiva and gingival recession (1). To correct soft-tissue defects around teeth and implants, grafts, important periodontal and implant plastic surgery procedures, are used (2,3).

Before performing periodontal plastic surgery, some fundamental principles must be taken into account during the preoperative phase, such as the control of factors that affect the patient (oral hygiene, cessation of smoking and control of any systemic disease), principles relevant in the flap phase (design, mobilisation, advancement, adaptation and stabilisation), and infection control in the postoperative phase (effective oral hygiene measures, antiseptic treatment and other medications) (4). Various surgical techniques are recommended, including subepithelial connective tissue grafts and free gingival grafts, which provide excellent results in terms of increasing the thickness of the gingival tissue and width of the attached gingiva (2-6).

Before reaching a clinical decision, clinicians should assess the thickness of the palatal mucosa (PM) as a potential donor site. The palatal mucosa at the level of the premolar is a common region selected for obtaining subepithelial connective tissue grafts due to its histology, anatomy, appropriate tissue thickness and low risk of damage to the palatal neurovascular bundle (7). Similarly, closure of the wound is crucial for periodontal healing, especially after regenerative procedures. The first postoperative week appears to be critical for maintaining wound stability (8). Potential complications include necrosis, pain, excessive bleeding, prolonged discomfort and infection (3,9).

Various biomaterials have been used to promote healing of the donor site after graft harvesting (10-12). Fibrin-rich plasma (L-PRF) is a platelet concentrate obtained from autologous blood. This fibrin network leads to increased cell migration and proliferation, thereby promoting efficient healing (5,9,10). Mechanical haemostats are only appropriate for patients with a properly functioning coagulation system to improve bleeding control after a surgical procedure. In addition, mechanical haemostats absorb multiple times their weight in fluids, promote platelet activation and aggregation, produce a matrix at the bleeding site and activate the extrinsic coagulation pathway. These agents can be used as the first choice due to their immediate availability and affordability (13-16).

Therefore, the purpose of this study was to evaluate early healing in the donor area of the palate following the placement of a collagen sponge and the use of L-PRF.

## Material and Methods

This study was a pre experimental comparative study carried out to evaluate the variation in thickness and early healing of the donor area of the palate following the placement of a collagen sponge and the use of L-PRF, and then a free gingival graft was collected from the palate according to the requirements of each patient.

Thirty patients were treated at the Watanabe Clinic and Cadena Dentists from September to December 2021 in Lima, Peru. Approval was obtained from the Ethics Committee of the San Juan Bautista Private University (N°891-2021-CIEI-UPSJB). The study protocol was in accordance with the Declaration of Helsinki of 1975, revised in Tokyo in 2004. All patients signed an informed consent form and were able to withdraw at any time during the study.

Patient selection

Thirty patients (11 men and 19 women, aged 28-40 years) presenting at least two Cairo Type I or II recession sites (17) with a maximum depth of 4 mm were selected for this study and cared for at the Watanabe Clinic and String.

The patient selection criteria were as follows:

Inclusion criteria

•Healthy patients (ASA I) over 18 years of age with indication for surgical treatment of soft tissue graft (connective graft).

•Dentate maxillary arch without pathology: crowding between the canine and the first molar, palatal flanges, palatal torus etc.

•Maxillary arch without fixed and/or removable appliances: brackets, fixed partial dentures that include the canine up to the first molar and removable partial dentures.

•Oral hygiene index ≤20% (O’Leary).

•Male or female.

Patients must have read, understood and signed an informed consent form.

Exclusion criteria

•Patients with periodontal diseases

•Patients who were pregnant or lactating.

•Patients who had previous mucogingival surgery treatment.

•Smoker.

•Patients under drug treatment that affects the periodontium: phenytoin or cyclosporine.

•Patients with coagulation disorders, taking corticosteroids, uncontrolled diabetes mellitus or any systemic disease for which periodontal surgery is contraindicated and healing may be compromised.

•Patients with contact hypersensitivity to related materials used in the study.

Patient preparation

All patients selected for the study underwent an initial hygienic phase of treatment, which consisted of supragingival scaling, prophylaxis and oral hygiene instruction performed by a periodontology (RA) specialist. After completing the hygienic phase, patients were able to present an oral hygiene index ≤15% with no gingival inflammation at the time of surgery.

Information registration

A transparent self-curing acrylic splint was made to standardise the measurements so that they could be performed in the same position and recorded at different times.

**Figure 1:**
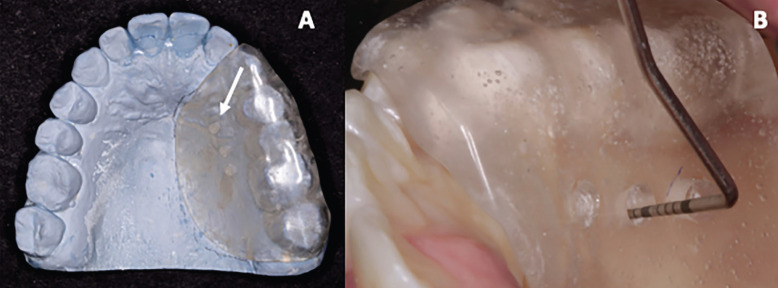
(A) Study model with the palatal acrylic splint. The initial reference point was made from the axis of the upper first premolar (3 mm from the gingival margin). (B) Assessment of palatal thickness using an acrylic palatal splint and a North Carolina periodontal probe.

Three holes were made in the splint with a 2-mm diameter high-speed round bur. The first reference point was located in the midpalatal area, 5 mm apical to the gingival margin of the first premolar. The second and third points were located 5 mm distant from the first point. For the measurement, a North Carolina probe was used, which was inserted perpendicular to the mucosal surface through the soft tissue with slight pressure until reaching a hard surface (bone tissue) (Fig. 1). These clinical measurements were performed by a trained clinical examiner (RA) and recorded pre- (baseline) and postsurgically (4 months later) in a file specifically prepared for this purpose.

In addition, based on clinical measurements, the healing process of the soft tissues (palate) was evaluated and recorded at 24 hours and 7, 14, 21 and 28 days after surgery using the Early Healing Index (EHI) and evaluation of re-epithelialisation. The EHI evaluates the closure of a wound according to epithelial proliferation and visible vascular neoformation. Re-epithelialisation was evaluated using the peroxide test with the presence or absence of bubbles and clinical signs according to the presence of fused margins, in contact, or presence of visible distance between the margins. Additionally, the presence of bleeding was evaluated after the first week in the margins of the incision. The thickness of the donor area of the palate was evaluated 4 months after surgery.

-Presurgical protocol

One hour before surgery, each patient received an intramuscular injection of ketorolac 60 mg + dexamethasone 4 mg, which was applied in the same manner the day after surgery. Likewise, 500 mg of amoxicillin was prescribed for 6 days, and pain was controlled with 500 mg paracetamol conditioned to pain.

-Surgical procedure

Before surgery, intravenous blood was collected in 10-mL glass-coated plastic tubes without anticoagulants. According to the manufacturer’s recommendations, the blood was immediately centrifuged at 13,000 rpm for 8 minutes using a Choukroun A L-PRF 12 centrifuge (France). After centrifugation, the fibrin clot was removed from the tube using sterile forceps, and adhering red blood cells were cut away with scissors. The fibrin clots were placed on a grid in the L-PRF-BOX, compressed with a cover to create a fibrin membrane and then placed in the required area. Extraoral asepsis was performed with gauze with iodine, and then intraoral asepsis was performed with 0.12% chlorhexidine solution (rinse). Surgical procedures were performed by a periodontology specialist trained in the free palate graft (RA) technique. To obtain grafts of similar size from each patient with wounds of similar characteristics and dimensions, the specialist underwent preclinical training using animal tissues, with the aim of removing a 15×6 mm graft with a uniform thickness of 2 mm, measured along the centre part by means of a calliper. Training was continued until the graft size (height, width and thickness) differed by no more than 5% in five consecutive samples.

The incision was extended from the line of the distal angle of the patient’s canine to the line of the mesial angle of the maxillary first molar. The most coronal horizontal incision, 15 mm long, was made 3 mm apical to the gingival margin; a second horizontal incision of the same length was made 6 mm from the first in a more apical position. Two vertical incisions were made to join the ends of the horizontal incisions and delimit the graft area. In this way, a partial thickness rectangular graft of 2 mm thickness was obtained. The graft was then measured. Subsequently, the fatty tissue was removed and de-epithelialized with a 15c scalpel blade and adapted to the required area or recipient area.

In Group A (n=15 patients), the palatal wound was covered by a quadruple layer of L-PRF, obtained after folding two L-PRF membranes upon themselves. In Group B (n=15 patients), the wound was covered with a collagen sponge (Hemotamp, Lima, Peru) as required by the patients. Both were sutured with horizontal crossed suspensory sutures (18).

-Postoperative procedure

Patients were instructed to rinse with chlorhexidine solution (0.12%) twice a day (morning and night) for 1 minute for 2 weeks to control plaque in the treated area. In case of pain, 500 mg paracetamol was prescribed conditioned to pain. Fourteen days after surgical treatment, the stitches were removed. After that period, patients were instructed to resume mechanical dental cleaning after each meal (3 times daily) with a soft-bristled toothbrush. All patients were included in a weekly periodontal maintenance program (professional plaque control) for the first 4 weeks and then monthly until the end of the study period.

**Figure 2:**
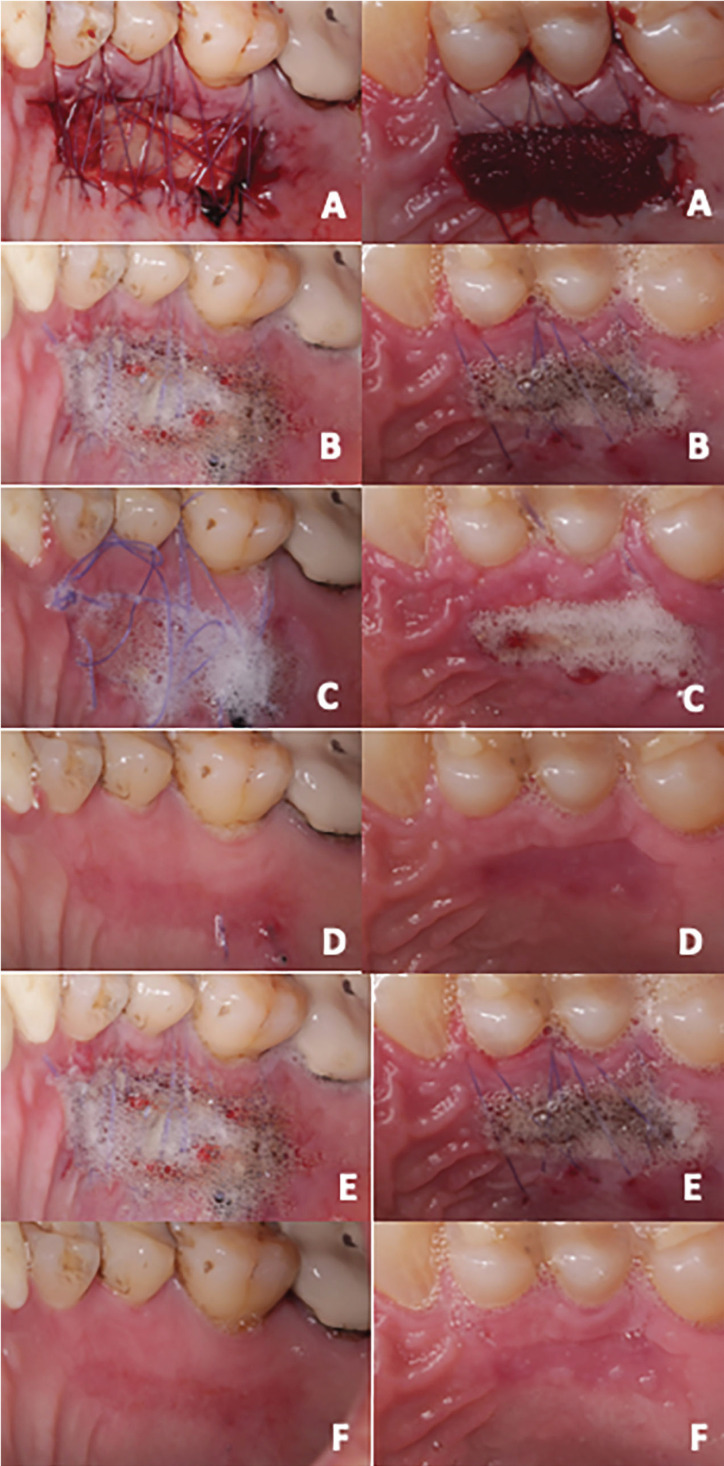
Comparison of palate areas between L-PRF (images on the left side) and EC (images on the right side) at 24 hours and 7, 14, 21 and 28 days postsurgery. A. Horizontal crossed suspensory sutures. B. Twenty-four hours after surgery, they used the peroxide test, in which the presence of bubbles was observed. C. Seven days after surgery, in addition to bubbles, clinical signs were observed according to the presence of fused margins. D. Twenty-one days after surgery. E. Twenty-eight days postsurgery.

Finally, postoperative controls were performed at 24 hours and 7, 14, 21 and 28 days, in which the healing process was observed in both study groups (Fig. 2).

## Results

**Figure 3:**
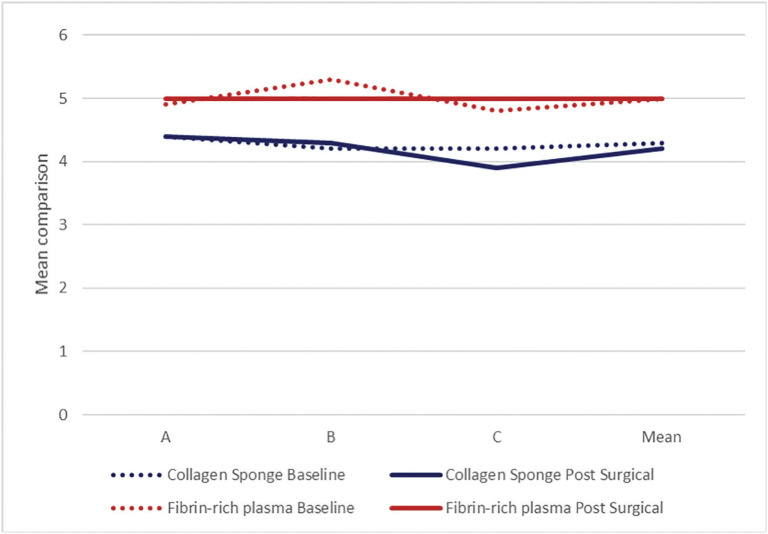
Average baseline and postsurgical palatal mucosa 4 months after application of collagen sponge and fibrin-rich plasma.

At baseline in the collagen sponge group, the palatal mucosa was between 1.7 and 7.0 mm, with a mean of 4.3 ± 1.4 (95.0% CI=(3.60–5.01)), and in the postsurgical evaluation at 4 months, the palatal mucosa was found to be between 2.3 and 7.0 mm, with a mean of 4.2 ± 1.3 (95% CI=(3.54–4.90)). In the L-PRF group, the palatal mucosa at baseline ranged from 3.0 to 7.7 mm, and in the postsurgical control, the palatal mucosa ranged from 3.0 to 7.0 mm; however, both controls coincided, with a mean of 5.0 mm (± 1.5 95% CI=(4.28–5.77) and ±1.2 95.0% CI=(4.43–5.61)), respectively (Table 1). In the L-PRF group, it was observed that the thickness of the palatal mucosa, without exception, decreased after the surgical intervention and then returned to its baseline condition 4 months later, whereas in the collagen sponge group, the gain of the palatal mucosa was less than the baseline measurement, with a difference of 0.1 mm (Fig. 3).

**Table 1:**
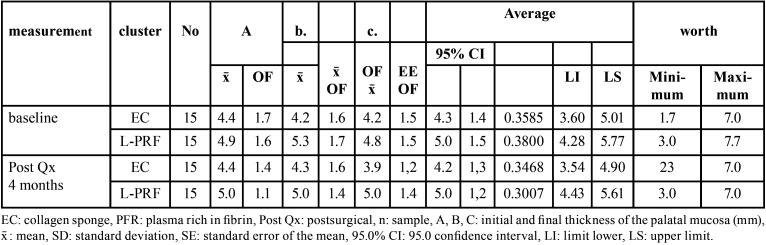
Descriptive values of baseline and postsurgical palatal mucosa 4 months after application of collagen sponge and fibrin-rich plasma.

The thickness of the palatal mucosa before and after postsurgical control (ӽMe-ӽF) in the collagen sponge (CE) group ranged from −1.0 to 1.3, with a mean difference of 0.1 ± 0.8 mm; 95% CI=(−0.341–0.518) (*p*=0.691). The L-PRF group ranged from −0.3 to 1.0, with a mean difference of 0.0 ± 0.5 mm; 95% CI =(−0.229–0.229); L-PRF achieved a gain in palatal mucosa equal in proportion to the baseline measurement (*p*=0.934). When comparing the collagen and fibrin-rich plasma groups, no significant difference was found (*p*=0.932) (Table 2).

**Table 2:**
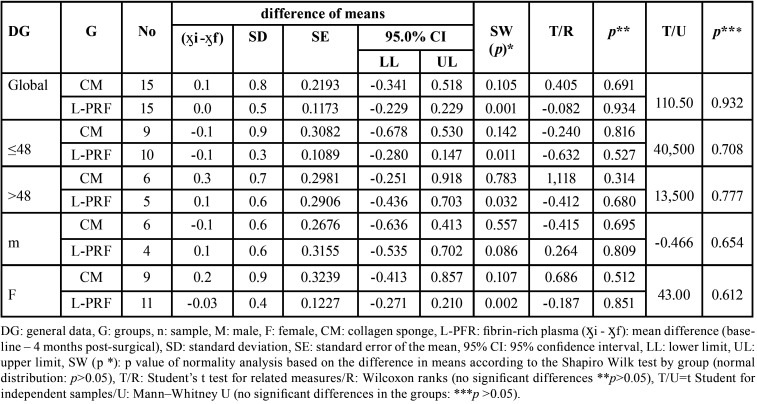
Differences in the means of the palatal mucosa at baseline and 4 months after the application of the collagen sponge and fibrin-rich plasma.

Participants under 48 years of age in both the CE and L-PRF groups presented greater palatal mucosa gain, with a mean difference before and after of −0.1 ± 0.9 mm (95% CI=(−0.678–0.530)) and −0.1 ± 0.3 mm (95% CI=(−0.280–0.147)), respectively, whereas in the participants older than 48 years, the palatal mucosa was greater in the baseline measurement, with a mean difference of 0.3 ± 0.7 (95% CI=(−0.251–0.918)) and 0.1 ± 0.6 mm (95% CI=(−0.436–0.703 )), respectively. These numerical differences according to age in both groups did not reach statistical significance (*p*=0.708 and *p*=0.777, respectively) (Table 2).

When CE was applied according to sex, a greater gain of palatal mucosa was found in men, with a mean difference of −0.1 ± 0.6 mm (95% CI=(−0.636–0.413)) compared to women, who had baseline gain of 0.2 ± 0.9 mm (95% CI=(−0.413–0.857)). This was in contrast to the L-PRF group, in which women achieved a greater gain of palatal mucosa (−0.03 ± 0.4 mm; 95% CI=(−0.271–0.210)) compared to men, with a baseline predominance of 0.1 ± 0.6 (95% CI=(−0.535–0.702)). However, when comparing the groups according to sex, no significant difference was found (*p*=0.654 and 0.612, respectively) (Table 2).

**Figure 4:**
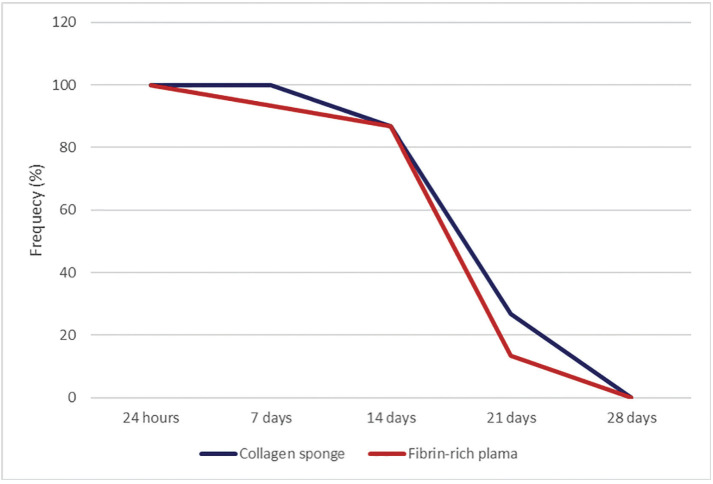
Percentage distribution of the presence of bubbles (peroxide test) in the palatal mucosa with collagen sponge application and fibrin-rich plasma.

The results of the hydrogen peroxide reactive test in both groups were positive in 100.0% of the discontinuity of the palatal mucosa at 24 hours, ceasing at 28 days. Both groups exhibited with a positive response to the test on Day 14, at 86.7%. Numerically, there was a downwards curve favourable to the fibrin-rich plasma group, in which the positive response decreased to 93.3% after 7 days; after 21 days, the positive response decreased to 13.3% compared to the collagen sponge group, which showed positive responses of 100.0% and 26.7%, respectively (Table 3, Fig. 4).

**Table 3:**
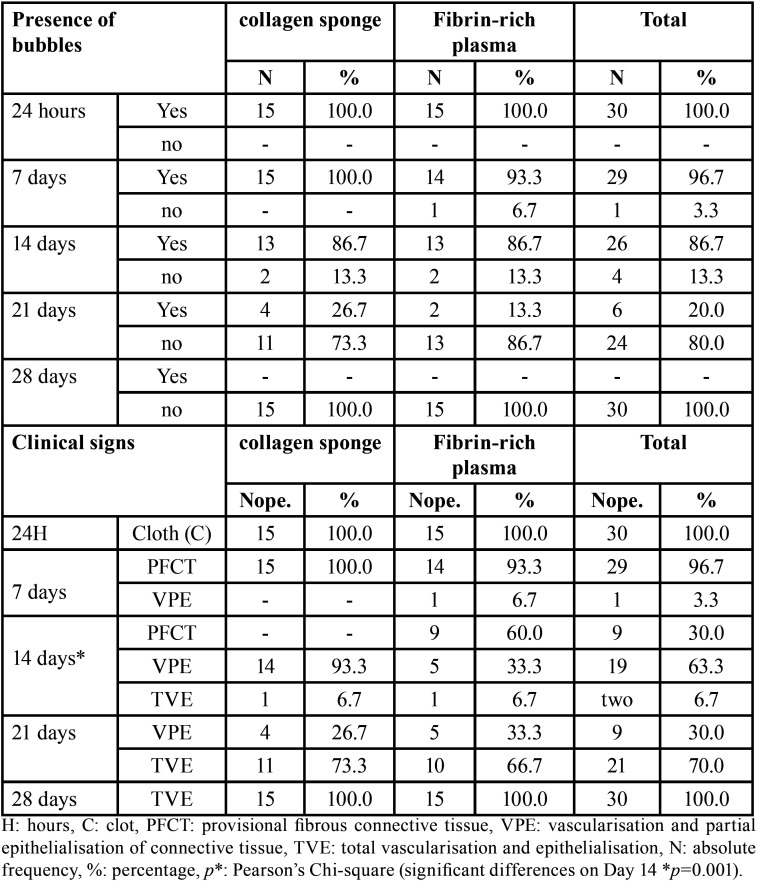
Re-epithelialisation of the palate mucosa according to the peroxide test at different times and rate of early healing of the palate mucosa in the groups with collagen sponge application and fibrin-rich plasma.

In the analysis of the healing index, it was found that at 24 hours, both groups presented clots in the solution of continuity of the palatal mucosa; on Day 28, they coincided in 100.0% of the cases with palatal mucosa with vascularisation and total epithelialisation. On Day 14, the clinical signs of vascularisation and partial epithelialisation of connective tissue predominated in the EC group at 93.3%, whereas in the L-PRF group, it was 33.3%; these numerical differences were statistically significant (*p*=0.001). On the other days, no significant differences were found (*p*>0.05); however, there was a favourable numerical predominance for the CD group, which on day 7 presented 100.0% provisional fibrous connective tissue, and on Day 21, 73.3% presented total vascularisation and epithelialisation (Table 3).

At 24 hours postsurgery, participants under 48 years of age presented a positive reactive test to hydrogen peroxide in 100.0% of cases in both groups; however, favourable decreases were observed in the L-PRF group, with 90.0%, 80.0%, and 10.0% reactive at 7, 14 and 21 days, respectively, and on Day 28, the EC and L-PRF groups both exhibited a negative response to the aforementioned test. On the other hand, at 24 hours and 7 days postsurgery, participants older than 48 years presented a positive reactive test in 100.0% of cases in both groups; however, a favourable decrease was observed in the CE group (66.7% and 16.7% reactive at 14 and 21 days, respectively), and on Day 28, both the EC and L-PRF groups exhibited a negative response to the hydrogen peroxide test.

According to sex, at 24 hours, both groups presented a positive reactive test for hydrogen peroxide in 100.0% of cases. Men in the L-PRF group presented a decrease in reactivity of 75.0% at 7 days, followed by 66.7% at 14 days in the EC group, and then the test became negative at 21 days in the L-PRF group. On Day 28, both groups exhibited a negative response. At 24 hours and 7 days, women in both groups presented a positive reactive test in 100.0% of cases; however, a favourable curve was observed for the L-PRF group, with decreases of 90.9% and 18.2% reactive at 14 and 21 days, respectively, and on Day 28, both the EC and L-PRF groups exhibited a negative response to the hydrogen peroxide test.

According to age, participants in the CE and PRF groups showed the presence of a clot in 100.0% of cases at 24 hours. Participants under 48 years of age in the CE group presented provisional fibrous connective tissue at 100.0% after 7 days; at 14 days, vascularisation and partial epithelialisation of connective tissue were observed in 100.0% and 20.0% of participants in the L-PRF group, and this numerical difference reached a statistical significance (*p*=0.000). At Day 21 in the L-PRF group, 70.0% vascularisation and total epithelialisation predominated, coinciding on Day 28 in both groups, with 100.0% of palatal mucosa with total vascularisation and epithelialisation. Participants older than 48 years in both groups presented provisional fibrous connective tissue in 100.0% of cases at 7 days.

At 24 hours, male participants in both groups presented clots in the palatal mucosa in 100.0% of cases, and at 7 days in the CD group, provisional fibrous connective tissue predominated in 100.0% of cases. At 14 days, vascularisation and partial epithelialisation of connective tissue were observed in 83.3%, and at 21 days, vascularisation and total epithelialisation were observed in 100.0% of cases in the L-PRF group. On Day 28, 100.0% of cases with palatal mucosa in both groups exhibited vascularisation and total epithelialisation. In women in the CE and PFR groups at 24 hours and 7 days, predominated clot and provisional fibrous connective tissue were observed in 100.0% of cases, respectively. Vascularisation and partial epithelialisation of connective tissue was observed in 100.0% of cases in the CD group and 36.4% in the L-PRF group at 14 days; these numerical differences were statistically significant (*p*=0.013).

## Discussion

The palatal mucosa at the level of the premolars is one of the most commonly used areas of the oral cavity to obtain connective tissue grafts due to its thickness and the anatomical and histological characteristics it presents, which are considered the gold standard in periodontal plastic surgery. Grafts from this area generate better clinical and aesthetic results in the long term (1,15,16). In the present study, it was possible to observe that the palatal mucosa was between 1.7 and 7.0 mm, with a mean of 4.3 ± 1.4. These measurements were very similar to those reported in the study of Kiziltoprak and Uslu (19), who found 4.31 ± 0.38 when evaluating the thickness of the palatal mucosa. However, the measurements found in this study are higher than those found by Said *et al*. (20), who reported an overall mean thickness of the palatal masticatory mucosa of 3.23 ± 0.47 mm; Anuradha *et al*. (21), who reported a mean thickness of 2.0 mm to 3.7 mm; and Bertl *et al*. (22), who reported a range between 2.65 mm and 6.89 mm in terms of average thickness of the palatal mucosa; however, this study was carried out on human cadavers.

The objective of this study was to evaluate the early healing of the donor area of the palate following the placement of a collagen sponge and the use of L-PRF, from the moment of obtaining the palate graft until 28 days, with healing evaluated using the peroxide reagent test. This test is based on the principle that if the epithelium is discontinuous, H2O2 diffuses into the connective tissue, where the catalase enzyme acts on the H2O2 to release water and oxygen. This is clinically demonstrated by the production of bubbles in the wound. If bubbles appear, the surgical area is not completely epithelialised (9,21,23).

In this study, both groups presented clots in the solution of continuity of the PM at 24 hours, and on Day 28, 100.0% of the cases exhibited PM with total vascularisation and epithelialisation. However, on Day 14, the collagen sponge group presented a greater clinical sign of vascularisation and partial epithelialisation of connective tissue (93.3%) than the L-PRF group (33.3%). This differs from what was found by the study of Femminella *et al*. (9), in which the L-PRF group showed significantly faster epithelialisation compared to the collagen sponge group, and at the end of week 3, all the palatal wounds of the test patients were completely epithelialised. Patarapongsanti *et al*. (23) evaluated the healing of the extraction of the free gingival graft in the palatal donor areas after using platelet-rich fibrin and oxidized regenerated cellulose, and they concluded that both groups completed the epithelialisation process within a week (3). However, in our study, 100% epithelialisation occurred by the end of week 4.

The use of L-PRF as a treatment alternative can accelerate PM wound healing and reduce patient pain (24,25). This faster epithelialisation could be related to increased cell growth, fibroblast proliferation, myofibroblasts and the synthesis of type I collagen, which leads to faster healing (25).

In relation to age, the participants under the age of 48 years presented faster healing (epithelialisation) compared to participants over the age of 48; however, in both cases on Day 28 they exhibited a negative response to the hydrogen peroxide test, both in the collagen sponge group and the L-PRF group, which may have been due to the greater potential for cell regeneration in younger people than in older people (20).

Rocha *et al*. (24) evaluated the thickness of donor tissue after increasing the connective tissue in the palatal area by implanting a lyophilized collagen sponge, obtaining a significant increase in mean thickness of 1.08 mm 4 weeks after surgery. In the present study, a greater PM gain was found in men when collagen sponge was applied (0.1 ± 0.6 mm) compared to women (0.2 ± 0.9 mm), whereas in the L-PRF group, women had greater PM gain (−0.03 ± 0.4 mm) than men (0.1 ± 0.6). However, these numerical differences were not statistically significant. These numerical gains may be related to the characteristics of the collagen sponges, which are haemostatic and biocompatible and have a favourable effect on wound healing and blood coagulation, thereby facilitating wound maturation and stability by improving the initial formation of clots and fibrin bonds (24,25).

In conclusion, there was no statistically significant difference in the thickness of the palatal mucosa between the use of L-PRF or a collagen sponge. However, the collagen sponge showed faster healing than L-PRF in the processes of vascularisation and total epithelialisation. New investigations with larger, paired and equiTable samples are suggested.
